# Option Portfolio Selection with Generalized Entropic Portfolio Optimization

**DOI:** 10.3390/e22080805

**Published:** 2020-07-22

**Authors:** Peter Joseph Mercurio, Yuehua Wu, Hong Xie

**Affiliations:** 1 Department of Mathematics and Statistics, York University, Toronto, ON M3J 1P3, Canada; 2 Manulife Financial Corp, Toronto, ON M4W 1E5, Canada; hong_xie@manulife.com

**Keywords:** relative entropy, Kullback–Leibler divergence, portfolio optimization, portfolio selection, option portfolios, European options, credit spreads, straddles, strangles, iron condors

## Abstract

In this third and final paper of our series on the topic of portfolio optimization, we introduce a further generalized portfolio selection method called generalized entropic portfolio optimization (GEPO). GEPO extends discrete entropic portfolio optimization (DEPO) to include intervals of continuous returns, with direct application to a wide range of option strategies. This lays the groundwork for an adaptable optimization framework that can accommodate a wealth of option portfolios, including popular strategies such as covered calls, married puts, credit spreads, straddles, strangles, butterfly spreads, and even iron condors. These option strategies exhibit mixed returns: a combination of discrete and continuous returns with performance best measured by portfolio growth rate, making entropic portfolio optimization an ideal method for option portfolio selection. GEPO provides the mathematical tools to select efficient option portfolios based on their growth rate and relative entropy. We provide an example of GEPO applied to real market option portfolio selection and demonstrate how GEPO outperforms traditional Kelly criterion strategies.

## 1. Introduction

Our series on the topic of portfolio optimization has introduced novel entropy-based optimization problems that facilitate the selection of a variety of efficient portfolios. The return-entropy portfolio optimization (REPO) [1] allocates capital to an equity portfolio based on the expected return and Shannon entropy of portfolio returns. REPO was adapted to form the discrete entropic portfolio optimization (DEPO) [2] for application to portfolios comprised of assets with discrete distributed returns, like exotic instruments such as binary or digital options, or fixed-return options (FROs). In this paper we further extend DEPO to accommodate mixed returns with generalized entropic portfolio optimization (GEPO). GEPO can handle a combination of discrete and continuous returns, such as those exhibited by option strategies. Option strategies have expected return payoff functions that contain intervals of discrete returns and intervals of continuous returns.

The novel optimization methods introduced throughout our series on entropic portfolio optimization can be summarized as follows:

(1) Return-entropy portfolio optimization (REPO) [1]: for the selection of portfolios comprising continuous return assets, such as equities, indices, mutual funds, or other non-derivative financial products,

(2) Discrete entropic portfolio optimization (DEPO) [2]: for the selection of portfolios comprising strictly discrete return assets, such as binary options, digital options, fixed-return options (FRO), sports bets, or other betting wagers, and (3) Generalized entropic portfolio optimization (GEPO): for the selection of portfolios comprising assets with mixed returns (returns that can exhibit both continuous and discrete returns), like option strategies such as covered puts, married calls, credit spreads, straddles, long strangles, butterfly spreads, iron condors, and more. GEPO can handle portfolios that contain various types of options that are much more general than binary options. As seen in Section 2, these option strategies have a more generalized payoff function that include both continuous parts and discrete parts, and thus exist outside the scope of REPO or DEPO alone. GEPO can be thought of as combining the capabilities of both REPO and DEPO.

**Figure 1:**
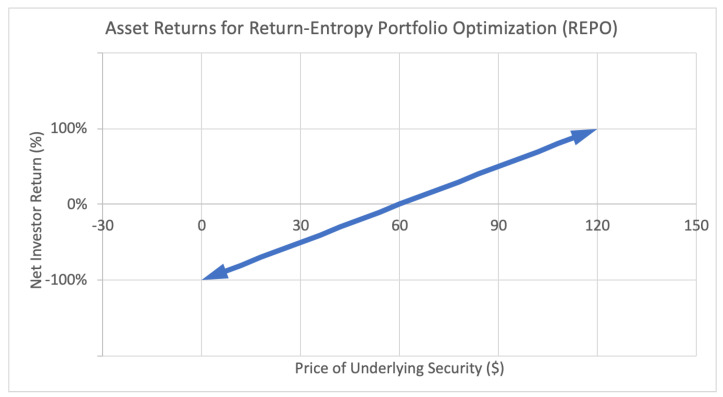
Sample asset return payoff function for class of assets used in return-entropy portfolio optimization (REPO).

The applications of these new optimization methods can be illustrated by the following investor return payoff function diagrams:

Figure 1 represents the payoff function for an investor with a regular long position in a stock. For every dollar increase or decrease in the underlying stock price, a proportional increase or decrease is generated in investor return. This payoff function is continuous, since the underlying stock price and thus investor returns fall on the (non-negative) real line.

**Figure 2:**
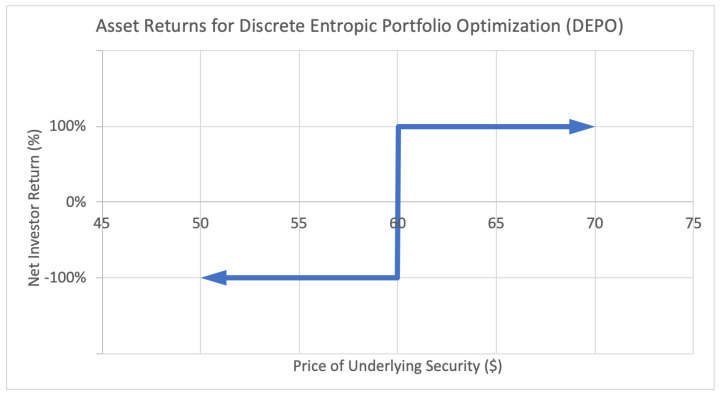
Sample asset return payoff function for class of assets used in discrete entropic portfolio optimization (DEPO).

The second Figure 2 illustrates the payoff function for a binary option. This type of option generates a fixed positive return *W* if the underlying asset price lands above a certain threshold, in this case a strike price of $60, and generates a fixed negative return *L* otherwise. This payoff function is strictly discrete, since the only possible return states are +W and −L.

**Figure 3:**
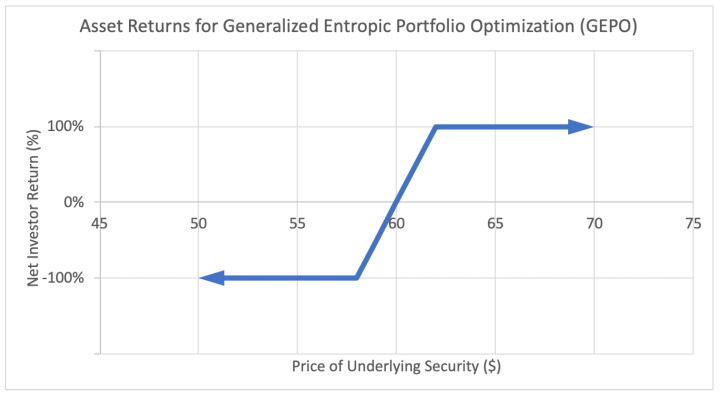
Sample asset return payoff function for class of assets used in generalized entropic portfolio optimization (GEPO).

The latter Figure 3, the focal topic of this paper, is an example of a payoff function for a more complex option strategy called a bull put credit spread. This strategy involves buying and selling put options (an option to sell assets at an agreed upon price and date) in such a manner to limit one’s potential risk. The resulting payoff function covers a “spread” outside of which the option behaves just like a binary option—it generates a fixed positive return *W* if the underlying asset price lands above the upper bound, and generates a negative fixed return *L* if it lands below the lower bound. In between, if the price lands between the boundaries of the spread, the investor receives a partial return, proportional to the distance between the midpoint and the underlying price, as seen in the payoff function. These returns can be both discrete and continuous: +W, −L, and anything in between. These kind of option strategies are the main motivation behind this paper.

Traditional portfolio optimization methods, such as those employed by Markowitz mean-variance portfolio optimization (MVPO) [3], are not suited to handle the discretely distributed nature of option returns as these distributions cannot be described by mean and variance alone. So continuing with the use of relative entropy as the proxy for portfolio risk (as used in DEPO), GEPO is an optimization method containing an objective function that simultaneously maximizes the portfolio growth rate and minimizes the relative entropy of the portfolio with respect to the uniform distribution. In the case of option portfolios, GEPO selects a collection of options from a set of possible choices, for example a portfolio of credit spreads, in order to maximize the expected growth rate for a given level of relative entropy risk. By using the combinatorial generating functions to empirically calculate entropy, as done in REPO and DEPO, we are able to calculate the relative entropy risk of an option portfolio in GEPO by extending the scope to include both discrete and continuous returns. GEPO calculates the relative entropy over multiple return probability states, including return states with continuous returns. In terms of the expected return, the Kelly criterion provides valuable insights into maximizing the portfolio growth rate of an option portfolio. To that effect, we extend the Kelly criterion growth rate to include instances of both discrete and continuous returns. GEPO empowers investors to quantitatively select a portfolio of options based on their risk–reward tolerance.

The remainder of this paper is organized as follows. The following Section 1.1 gives a literature review of research on the topic of option portfolio optimization. Section 2 explains the technical details behind maximizing the portfolio growth rate by extending the Kelly criterion to generalized option strategies, with specific examples for several popular option strategies. Section 3 provides a brief review of information theory, Shannon entropy and Kullback–Leibler divergence, the foundation of entropic portfolio optimization. As the main feature of this paper, the GEPO problem is presented in Section 4. Finally, Section 5 demonstrates an example of GEPO selecting a portfolio of equity credit spreads chosen from the S&P100 composite index, and shows how GEPO outperformed the Kelly criterion and alternative Kelly criterion methods.

### 1.1. Literature Review

Option portfolio optimization is a considerably new topic of research, with the earliest found work beginning only this millennium. For a constant relative risk aversion investor, Liu (2003) [4] modeled the stochastic volatility to optimize a portfolio comprising one equity, a put option, and cash, deriving an analytic solution for the optimal allocation. Unfortunately for this method, a parametric model must be specified and the risk is mostly concentrated on just one option. Jones (2006) [5] exploited apparent mispricing of put options to derive an optimal portfolio of options using a general nonlinear latent factor model, but this model is overburdened by numerous required parameters. Eraker (2007) [6] modeled stochastic volatility parametrically, and then used the traditional mean-variance framework to optimize allocation between straddles, puts, and calls, yielding a closed-form solution for portfolio weights. Haugh (2007) [7] used duality and approximate dynamic programming (ADP) methods to facilitate high-dimensional American option pricing and portfolio optimization. Zymler (2011) [8] used robust portfolio optimization aimed to maximize the worst-case portfolio return for designing portfolios that include European-style options. This model trades off weak and strong guarantees on the worst-case portfolio return.

A moderate amount of research into option portfolio optimization has been conducted with respect to the use of value-at-risk (VaR) or conditional value-at-risk (CVaR) as a risk measure. See Alexander (2003, 2006) [9,10], Zymler (2013) [11], and Maasar (2016) [12]. As illustrated by Alexander, the VaR and CVaR minimization problems for derivative portfolios are typically ill-posed in the sense that there are many portfolios that have similar CVaR/VaR values to that of the optimal portfolio and slight perturbation of the data can lead to significantly different optimal solutions. Zymler notes that optimization problems involving VaR are often computationally intractable and require complete information about the return distribution of the portfolio assets, which is rarely available in practice. For these reasons, we conclude that VaR and CVaR methods are not ideal approaches to option strategy portfolio optimization.

Driessen (2013) [13] used generalized method of moments to maximize expected returns for a portfolio comprising a stock, an option strategy (puts and straddles), and cash. Constantinides (2013) [14] leverage-adjusted portfolios of either calls or puts by using the *omega* or *lambda*, known as the options’ elasticity. Fadugba (2014) [15] used a binomial model as a performance measure to price American and European options, and exploited mispricings to derive optimal portfolios. In a continuous time regime-switching market, Fu (2014) [16] introduced a functional operator to maximize the expected utility of the terminal wealth of a portfolio that contains an option, an underlying stock and a risk-free bond. Fatyanova (2017) [17] developed a constrained optimization problem for constructing an option portfolio that maximizes a certain payoff function. Faias (2017) [18] also noted that traditional portfolio optimization methods like mean-variance optimization are not suitable for option portfolios due to non-normality and difficulty estimating distribution of returns, then introduced a short-term view objective function used to optimize portfolios of European options mainly by exploiting mispricing between options. Zhao (2018) [19] used first- and second-order moments to model options returns and extended the Markowitz mean-variance framework to include option selection with much lower computational time than off-the-shelf solvers. Zeng (2018) [20] introduced a progressive hedging algorithm using reinforcement learning (Q-learning) for option portfolio optimization that was first of its kind to consider the exercise timings of an American option.

Almost all these alternative methods require some kind of parametric model assumption and are limited to the number of options composing the portfolio. GEPO is non-parametric and delivers well-diversified portfolios of many options with no limit. Additionally, all these methods misguidedly aim to maximize the portfolio expected returns, and besides the use of VaR and CVaR there has not yet been any suggestion for measuring or managing the risk of these option portfolios. GEPO not only minimizes the relative entropy risk of the portfolio, but also maximizes the proper metric for measuring the future performance of option returns: exponential growth rate.

## 2. Maximum Exponential Growth Rate

### 2.1. The Kelly Criterion for Multiple Wagers

As previously presented in Mercurio et al. (2020) [2], we use the extension of the Kelly criterion (Kelly, 1956) [21] to *n* wagers. For events $Y_{1},\ldots ,Y_{n}$ with success probabilities $p_{1},\ldots ,p_{n}$, let *w* represent the total percentage of bankroll to be wagered. For a portfolio that allocates the wager equally across among events, the growth rate coefficient *G* would be

(1) $$G\left(w\right)=\left(\frac{1}{n}\sum_{i=1}^{n}p_{i}\right)\log\left(1+w\right)+\left(1-\frac{1}{n}\sum_{i=1}^{n}p_{i}\right)\log\left(1-w\right),$$

Therefore, by denoting $\bar{p}=\frac{1}{n}\sum_{i=1}^{n}p_{i}$ (this can be thought of as a blended probability of success), the Kelly criterion can be used here to identify the optimal size wager for maximum growth rate as $w^{*}=2\bar{p}-1$.

### 2.2. Extension of the Kelly Criterion to Option Strategies

This section is a brief review of several most popular option strategies, with a summary of the details and motivation behind each. Additionally, we extend the Kelly criterion to find optimal growth rates of each option strategy. Further details and strategies can be found in the TMX Group Montreal Exchange guides and strategies documentation (2020) [22].

#### 2.2.1. Covered Call

**Figure 4:**
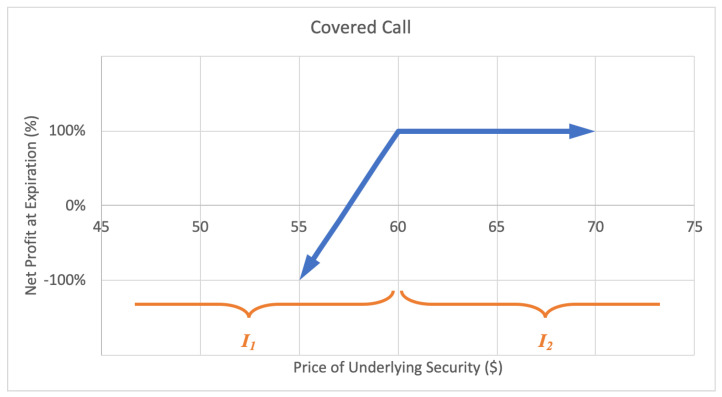
Sample expected payoff function for a covered call strategy.

A covered call option strategy involves selling a call that is covered by an equivalent long stock position, providing a hedge on the stock. In exchange for upside potential, an investor can earn income on the premium. The motivation behind a covered call is to earn premium income. The maximum loss is limited to the initial stock purchase price slightly reduced by the premium income from sale of the call option. The maximum gain is limited, equal to the strike price less the initial stock purchase price, plus the premium income received. A sample expected payoff function for a covered call strategy is illustrated below in Figure 4.

For the price of underlying security *S* and option return *R*, the growth rate for a covered call strategy becomes

(2) G(w)=ϱlog(1+αw)+plog(1+w),

where ϱ represents the probability $P\left(S\in I_{1}\right)=P\left(R<1\right)$, $p_{2}$ represents the probability $P\left(S\in I_{2}\right)=P\left(R=1\right)$ with p+ϱ=1, and α is the expected value of returns conditional on underlying price falling on interval $I_{1}$, $\alpha =E(R\;|\;S\in I_{1})$. Then *G* is maximized by differentiating with respect to *w* and setting equal to zero,

(3) $$\begin{array}{cc} \frac{\partial }{\partial w}G & =\frac{p}{1+w}+\frac{\alpha \varrho }{1+\alpha w}\\ \Rightarrow 0 & =p\left(1+\alpha w^{*}\right)+\alpha \varrho \left(1+w^{*}\right)\\ \Rightarrow w^{*} & =\frac{p(\alpha -1)}{\alpha }-1, \end{array}$$

which implies a positive $w^{*}$ exists if and only if p>α/(α−1), for α≠0.

#### 2.2.2. Married Put

**Figure 5:**
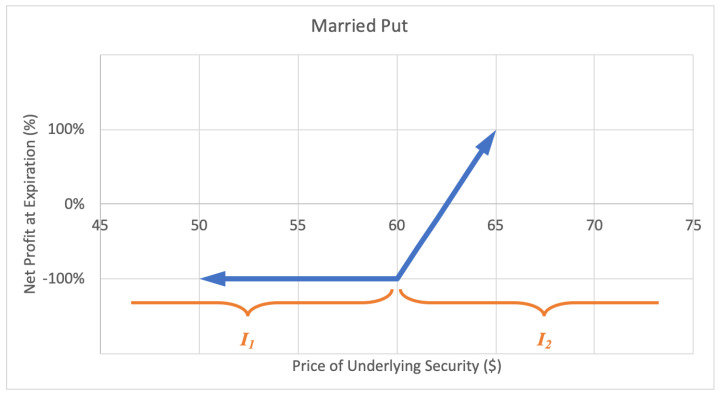
Sample expected payoff function for a married put strategy.

A married put, or protective put option strategy involves adding a long-put position to a long stock position, forming a lower bound for the stock value. The investor profits as the stock price keeps rising. The motivation for a married put is to hedge against a temporary decrease in the stock price. The maximum loss is the stock purchase price less the strike price of the put plus the premium paid for the option. There is unlimited potential gain for a married put. A sample expected payoff function for a married put strategy is illustrated below in Figure 5.

For the price of underlying security *S* and option return *R*, the growth rate for a married put strategy becomes

(4) G(w)=qlog(1−w)+ϱlog(1+αw),

where *q* represents the probability $P\left(S\in I_{1}\right)=P\left(R=-1\right)$, ϱ represents the probability $P\left(S\in I_{2}\right)=P\left(R>-1\right)$ with q+ϱ=1, and α is the expected value of returns conditional underlying price landing in interval $I_{2}$, $\alpha =E(R\;|\;S\in I_{2})$. Then *G* is maximized by differentiating with respect to *w* and setting equal to zero,

(5) $$\begin{array}{cc} \frac{\partial }{\partial w}G & =\frac{\alpha \varrho }{1+\alpha w}-\frac{q}{1-w}\\ \Rightarrow 0 & =\alpha \varrho \left(1-w^{*}\right)-q\left(1+\alpha w^{*}\right)\\ \Rightarrow w^{*} & =1-\frac{q(\alpha +1)}{\alpha }, \end{array}$$

which implies a positive $w^{*}$ exists if and only if q<α/(α+1), for α≠0.

#### 2.2.3. Credit Spread

**Figure 6:**
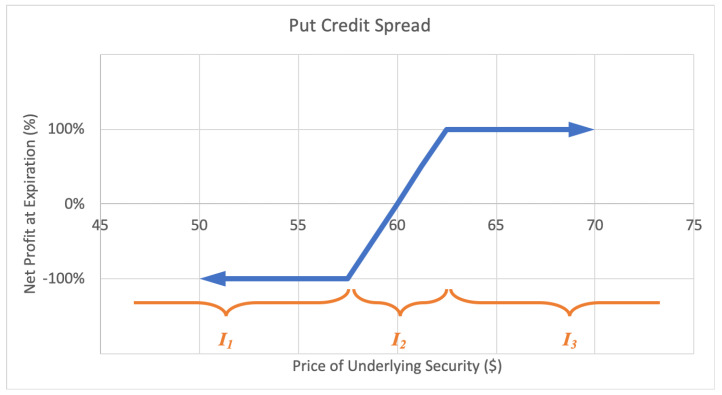
Sample expected payoff function for a put credit spread strategy.

A put credit spread, or bull put spread option strategy consists of a short-put option at a certain strike price and a long-put option at lower strike price. The investor profits with a rise in the underlying stock price. The motivations for a credit spread include to earn income with limited risk and to moderately profit from a rise in the stock price. The maximum loss is limited, equal to the net difference between the higher and lower strike prices less the net premium received. The maximum gain too is limited to the net premium received when putting on the position. A sample expected payoff function for a put credit spread strategy is illustrated below in Figure 6.

**Figure 7:**
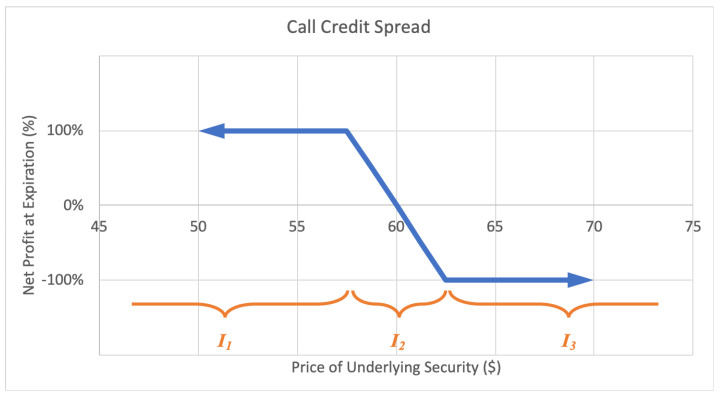
Sample expected payoff function for a call credit spread strategy.

Alternatively, a call credit spread, or bear call spread option strategy consists of a short call option at a certain strike price and a long call option at a higher strike price. This way the investor profits with a decrease in the underlying stock price. The maximum loss is limited, equal to the net difference between the higher and lower strike prices less the net premium received. The maximum gain too is limited to the net premium received when calling on the position. A sample expected payoff function for a put credit spread strategy is illustrated below in Figure 7.

For the price of underlying security *S* and option return *R*, the growth rate for a credit spread strategy becomes

(6) G(w)=plog(1+w)+ϱlog(1+αw)+qlog(1−w),

where *p* is the probability P(R=1), ϱ represents the probability $P\left(S\in I_{2}\right)=P\left(-1<R<1\right)$, *q* represents the probability P(R=−1), with p+q+ϱ=1, and α is the expected value of returns conditional on underlying price landing in interval $I_{2}$, $\alpha =E(R\;|\;S\in I_{2})$. Then *G* is maximized by differentiating with respect to *w* and setting equal to zero,

(7) $$\begin{array}{cc} \frac{\partial }{\partial w}G & =\frac{p}{1+w}+\frac{\alpha \varrho }{1+\alpha w}-\frac{q}{1-w}\\ \Rightarrow 0 & =p\left(1-w^{*}\right)\left(1+\alpha w^{*}\right)+\alpha \varrho \left(1+w^{*}\right)\left(1-w^{*}\right)-q\left(1+w^{*}\right)\left(1+\alpha w^{*}\right)\\ \Rightarrow w^{*} & =\frac{\left(\alpha p-\alpha q-p-q\right)+\sqrt{{\left(\alpha p-\alpha q-p-q\right)}^{2}+4\alpha \left(p-q+\alpha -\alpha p-\alpha q\right)}}{2\alpha }, \end{array}$$

by the quadratic formula, for α≠0.

#### 2.2.4. Straddle

**Figure 8:**
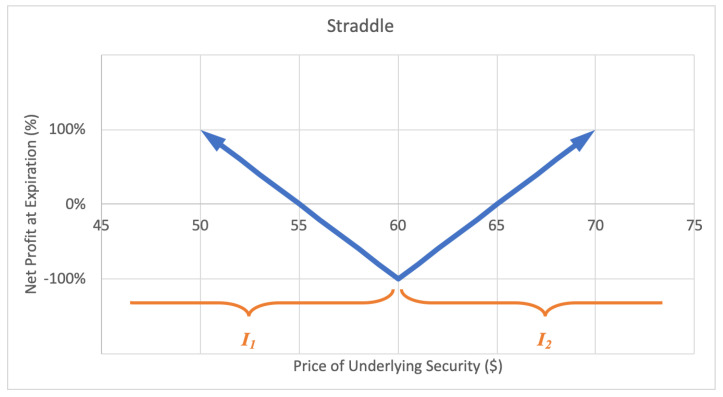
Sample expected payoff function for a straddle strategy.

A straddle option strategy involves buying a call and buying a put with equal strike price and expiration date. The investor profits when the underlying stock price experiences a big move up or down. The motivation behind a straddle is to capitalize on correctly predicting a big price move or high volatility in the near future. The maximum loss for a straddle is limited to the premium paid for the call and put options. The potential gain is unlimited. A sample expected payoff function for a straddle strategy is illustrated below in Figure 8.

For the price of underlying security *S* and option return *R*, the growth rate for a straddle strategy becomes

(8) G(w)=ςlog(1−βw)+ϱlog(1+αw),

where ς represents the probability $P(S\in I_{1})$, ϱ represents the probability $P(S\in I_{2})$, and expected values $\beta =-E(R\;|\;S\in I_{1})$ and $\alpha =E(R\;|\;S\in I_{2})$, while ϱ+ς=1. Then *G* is maximized by differentiating with respect to *w* and setting equal to zero,

(9) $$\begin{array}{cc} \frac{\partial }{\partial w}G & =\frac{\alpha \varrho }{1+\alpha w}-\frac{\beta \varsigma }{1-\beta w}\\ \Rightarrow 0 & =\alpha \varrho \left(1-\beta w^{*}\right)-\beta \varsigma \left(1+\alpha w^{*}\right)\\ \Rightarrow w^{*} & =\frac{\varrho }{\beta }+\frac{\varrho -1}{\alpha }, \end{array}$$

which implies a positive $w^{*}$ exists if and only if the odds ratio ϱ/(1−ϱ)>β/α, for α,β≠0.

#### 2.2.5. Long Strangle

**Figure 9:**
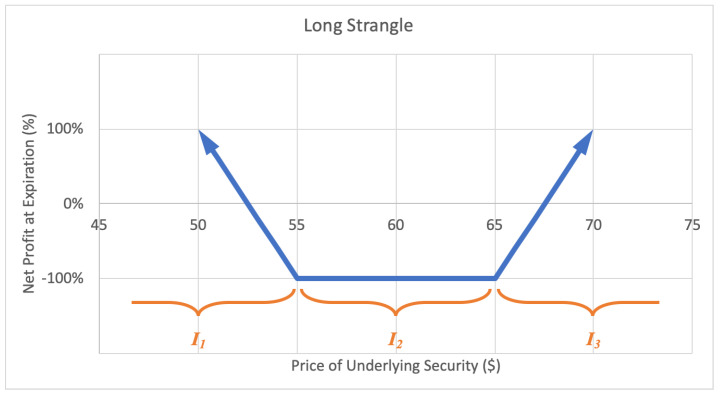
Sample expected payoff function for a long strangle strategy.

A long strangle option strategy involves buying an out-of-the-money call option and an out-of-the-money put option with the same expiration date. A strangle is similar to a straddle except a straddle has equal strike price whereas a strangle has a call option with higher strike price than the put option. The investor profits when there is a very big move up or down in the stock price. The motivation behind a long strangle is to capture a big move in the stock price over the term of the option. The maximum loss for a straddle is limited to the net premium paid for the call and put options. A sample expected payoff function for a long strangle strategy is illustrated below in Figure 9.

For the price of underlying security *S* and option return *R*, the growth rate for a long strangle strategy becomes

(10) G(w)=ςlog(1−βw)+qlog(1−w)+ϱlog(1+αw),

where ς represents the probability $P(S\in I_{1})$, *q* represents the probability $P\left(S\in I_{2}\right)=P\left(R=-1\right)$, and ϱ represents the probability $P(S\in I_{3})$, and expected values $\beta =-E(R\;|\;S\in I_{1})$ and $\alpha =E(R\;|\;S\in I_{3})$, while q+ϱ+ς=1. Then *G* is maximized by differentiating with respect to *w* and setting equal to zero,

(11) $$\begin{array}{cc} \frac{\partial }{\partial w}G & =\frac{\alpha \varrho }{1+\alpha w}-\frac{q}{1-w}-\frac{\beta \varsigma }{1-\beta w}\\ \Rightarrow 0 & =\alpha \varrho \left(1-w^{*}\right)\left(1-\beta w^{*}\right)-q\left(1+\alpha w^{*}\right)\left(1-\beta w^{*}\right)-\beta \varsigma \left(1+\alpha w^{*}\right)\left(1-w^{*}\right)\\ \Rightarrow w^{*} & =\frac{-\left(\alpha \beta q-\alpha \beta -\beta \varrho +\beta -\alpha q-\alpha \varrho \right)-\sqrt{{\left(\alpha \beta q-\alpha \beta -\beta \varrho +\beta -\alpha q-\alpha \varrho \right)}^{2}-4\alpha \beta \left(\beta q+\beta \varrho -\beta +\alpha \varrho -q\right)}}{2\alpha \beta }, \end{array}$$

by the quadratic formula, for α,β≠0.

#### 2.2.6. Butterfly Spread

**Figure 10:**
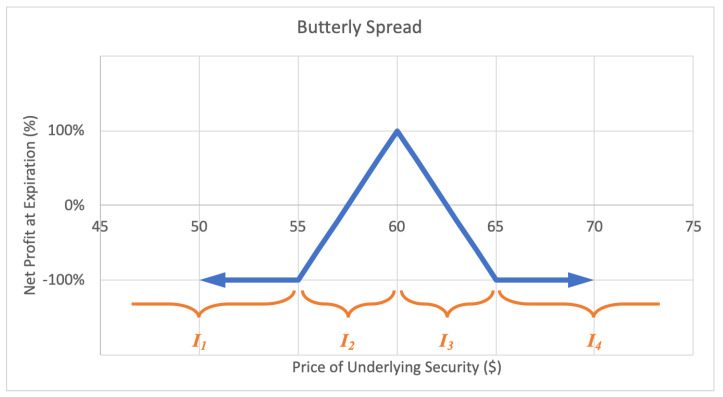
Sample expected payoff function for a butterfly spread strategy.

A butterfly spread, or long call butterfly option strategy consists of two short calls at a middle strike price and two long calls, one at the lower strike and one at the higher strike price, all with the same expiration date. The investor profits by correctly predicting the underlying stock price at expiration. The motivation behind a butterfly spread is to capitalize from predicting a target stock price at the options expiry date. The maximum loss for a butterfly spread is the short call strike price less the lower long call strike price less the net premium paid. The potential gain is unlimited. A sample expected payoff function for a butterfly spread strategy is illustrated below in Figure 10.

For the price of underlying security *S* and option return *R*, the growth rate for a butterfly spread strategy becomes

(12) $$G\left(w\right)=\frac{1}{2}q\log\left(1-w\right)+\varrho \log\left(1+\alpha w\right)+\varsigma \log\left(1-\beta w\right)+\frac{1}{2}q\log\left(1-w\right),$$

where *q* represents the probability $P\left(S\in I_{1}\cup I_{4}\right)=P\left(R=-1\right)$, ϱ represents the probability $P(S\in I_{2})$, ς represents the probability $P(S\in I_{3})$, and expected values $\alpha =E(R\;|\;S\in I_{2})$ and $\beta =-E(R\;|\;S\in I_{3})$, while q+ϱ+ς=1. Then *G* is maximized by differentiating with respect to *w* and setting equal to zero,

(13) $$\begin{array}{cc} \frac{\partial }{\partial w}G & =\frac{\alpha \varrho }{1+\alpha w}-\frac{q}{1-w}-\frac{\beta \varsigma }{1-\beta w}\\ \Rightarrow 0 & =\alpha \varrho \left(1-w^{*}\right)\left(1-\beta w^{*}\right)-q\left(1+\alpha w^{*}\right)\left(1-\beta w^{*}\right)-\beta \varsigma \left(1+\alpha w^{*}\right)\left(1-w^{*}\right)\\ \Rightarrow w^{*} & =\frac{-\left(\alpha \beta q-\alpha \beta -\beta \varrho +\beta -\alpha q-\alpha \varrho \right)-\sqrt{{\left(\alpha \beta q-\alpha \beta -\beta \varrho +\beta -\alpha q-\alpha \varrho \right)}^{2}-4\alpha \beta \left(\beta q+\beta \varrho -\beta +\alpha \varrho -q\right)}}{2\alpha \beta }, \end{array}$$

by the quadratic formula, for α,β≠0.

#### 2.2.7. Iron Condor

**Figure 11:**
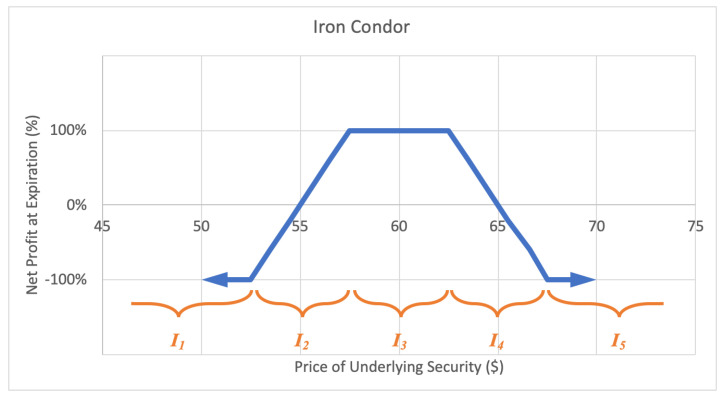
Sample expected payoff function for an iron condor strategy.

An iron condor, or short condor option strategy involves selling one call and buying another call with a higher strike price, plus selling one put and buying another put with a lower strike price, with the current underlying price falling between the call and put strikes. The investor profits if the underlying stock price is between the call and put strikes at option expiration. The motivation behind an iron condor is when the investor foresees the stock trading in a narrow range over the life of the options. The maximum loss of an iron condor is the greater of the difference between high and low call strikes and high and low put strikes, less the net premium received. A sample expected payoff function for an iron condor strategy is illustrated below in Figure 11.

For the price of underlying security *S* and option return *R*, the growth rate for an iron condor strategy becomes

(14) $$G\left(w\right)=\frac{1}{2}q\log\left(1-w\right)+\varrho \log\left(1+\alpha w\right)+p\log\left(1+w\right)+\varsigma \log\left(1-\beta w\right)+\frac{1}{2}q\log\left(1-w\right),$$

where *q* represents the probability $P\left(S\in I_{1}\cup I_{5}\right)=P\left(R=-1\right)$, ϱ represents the probability $P(S\in I_{2})$, *p* represents the probability $P\left(S\in I_{3}\right)=P\left(R=1\right)$, ς represents the probability $P(S\in I_{4})$, and expected values $\alpha =E(R\;|\;S\in I_{2})$ and $\beta =-E(R\;|\;S\in I_{4})$, while p+q+ϱ+ς=1. Then *G* is maximized by differentiating with respect to *w* and setting equal to zero,

(15) $$\begin{array}{cc} \frac{\partial }{\partial w}G & =\frac{p}{1+w}-\frac{q}{1-w}+\frac{\alpha \varrho }{1+\alpha w}-\frac{\beta \varsigma }{1-\beta w}\\ \Rightarrow w^{*} & =\frac{-\left(\alpha q-\alpha p+p+q\right)+\sqrt{{\left(\alpha q-\alpha p+p+q\right)}^{2}-4\alpha \left(\alpha p+\alpha q-\alpha -p+q\right)}}{2\alpha }, \end{array}$$

if and only if α=−β≠0 (reflective symmetry for expected values), by the quadratic formula.

## 3. Minimum Relative Entropy

For the purposes of GEPO, the risk of an option portfolio is defined here as the relative entropy of portfolio returns, with respect to the uniform distribution. In order to calculate the relative entropy, we first must calculate the Shannon entropy.

### 3.1. Shannon Entropy

As a quick review of information theory (Shannon, 1948) [23,24], the Shannon entropy of a random variable represents the amount of randomness inherent to that variable. For a discrete random variable *X* with probability mass function P(·) that can take on possible values $x_{1},\ldots ,x_{n}$, the Shannon entropy *H* is the average amount of information produced by *X*, defined as

(16) $$H\left(X\right)=E\left(-\log P\left(X\right)\right)=-\sum_{k=1}^{m}P\left(x_{k}\right)\log P\left(x_{k}\right).$$

For *n* discrete random variables $X_{1},\ldots ,X_{n}$ respectively with $m_{1},\ldots ,m_{n}$ states, the joint entropy of $X=(X_{1},\ldots ,X_{n})$ is given by

(17) $$H\left(X_{1},\ldots ,X_{n}\right)=-\sum_{k_{1}=1}^{m_{1}}\cdots \sum_{k_{n}=1}^{m_{n}}P_{X}\left(x_{1k_{1}},\ldots ,x_{nk_{n}}\right)\log P_{X}\left(x_{1k_{1}},\ldots ,x_{nk_{n}}\right).$$

This can be calculated empirically given a set of historical data by using the method introduced in REPO [1]. For the case of options returns, many strategies have more than just two (binary) states. Generally there can be up to four total states: positive discrete returns (+1), negative discrete returns (−1), continuous returns positively correlated with underlying price, and continuous returns negatively correlated with underlying price.

### 3.2. Kullback–Leibler Divergence

Kullback and Leibler (1951) [25,26] introduced the Kullback–Leibler divergence which measures the directed divergence between two probability distributions. For discrete probability distributions *P* and *Q*, the Kullback–Leibler divergence between *P* and *Q*, also known as the relative entropy of *P* with respect to *Q*, is given by

(18) $$\begin{array}{cc} D_{KL}\left(P\;∥\;Q\right) & =-\sum_{x\in \chi }P\left(x\right)\log\left(\frac{Q(x)}{P(x)}\right)\\ & =\;\sum_{x\in \chi }P\left(x\right)\log\left(\frac{P(x)}{Q(x)}\right). \end{array}$$

As shown in our previous paper (2020) [2], relative entropy qualifies as a convex risk measure based on the relative entropy principle. We once again use this quantity as the discriminatory risk measure for option portfolio optimization. For *m* total possible states, we will use the *m*-state discrete uniform distribution $U_{m}$ as the reference distribution and measure from there the distance to the distribution of portfolio returns. Thus, for Shannon entropy H(·), the risk of an option strategy portfolio $R_{Q}$ is measured by the relative entropy of $R_{Q}$ with respect to the uniform distribution $U_{m}$,

(19) $$D_{KL}\left(R_{Q}\;∥\;U_{m}\right)=\log\left(m\right)-H\left(R_{Q}\right).$$

Using the same combinatorial technique employed for REPO [1] and DEPO [2], the Shannon entropy of option portfolio returns $R_{Q}$ can be estimated empirically via probability generating functions. For a collection of *n* discrete return assets over time period j=1,…,T, let $r_{j}=\left(r_{1j},\ldots ,r_{nj}\right)$ denote the cross-sectional *n*-dimensional vector of outcomes across one observational row of data, and let them be uniquely represented by the collection of $u_{k}$’s such that $u_{k}=\left\{r_{j}\;|\;r_{j}\neq u_{l},\mathrm{for}\;\mathrm{some}\;j,\;\mathrm{and}\;\mathrm{any}\;l\neq k\right\}$. Then the empirical Shannon entropy of option portfolio returns $R_{Q}$ can be expressed as

(20) $$H\left(R_{Q}\right)\approx -\sum_{k=1}^{m}\left(\frac{g^{\left(k\right)}\left(0\right)}{k!}\right)\log\left(\frac{g^{\left(k\right)}\left(0\right)}{k!}\right),$$

for *k*th-derivative at x=0 of generating function

(21) $$g\left(x;w_{1},\ldots ,w_{n}\right)=\frac{1}{T}\sum_{j=1}^{T}x^{\left\{k\;:\;r_{j}=u_{k}\right\}}.$$

Therefore, the risk of an option portfolio is given by the relative entropy of portfolio returns $R_{Q}$, estimated empirically as

(22) $$D_{KL}\left(R_{Q}\;∥\;U_{m}\right)\approx \log\left(m\right)+\sum_{k=1}^{m}\left(\frac{g^{\left(k\right)}\left(0\right)}{k!}\right)\log\left(\frac{g^{\left(k\right)}\left(0\right)}{k!}\right),$$

for *m*-state discrete uniform distribution $U_{m}$.

## 4. Option Portfolio Selection Based on Growth Rate and Relative Entropy

### 4.1. Generalized Entropic Portfolio Optimization (GEPO)

The new generalized entropic portfolio optimization (GEPO) problem uses a multi-objective function that minimizes estimated relative entropy and maximizes expected growth rate. Using this optimization, investors can make portfolio selections based on a chosen risk tolerance. The highest risk portfolio solely maximizes the expected portfolio growth rate, equivalent to the Kelly criterion method. The lowest risk portfolio minimizes the portfolio relative entropy, the most diversified portfolio allocating capital to all *n* options equally. Somewhere in between lies a user’s optimal portfolio of choice. For the case of option strategies the returns can be both discrete and continuous in nature. For example, credit spread returns can be either +100% for a success, −100% for a failure, or somewhere in between −100% and +100% on a continuous scale for partial returns which we will denote by 0. Thus, we would have discrete return outcomes *u* such that u∈{−1,0,+1}. This leads to the generalized entropic portfolio optimization problem. Consider *n* potential option strategy contracts. Let λ be the number of probability states in the single-asset option strategy. For the generalized option strategy, returns can exhibit at most four general unique probability states: +100%,−100%, some continuous return on a positively sloped leg (with mean α), and some continuous return on a negatively sloped leg (with mean β), therefore 1<λ≤4. Let the up-to-four probability states be represented respectively by $p_{i},q_{i},\varrho_{i},\varsigma_{i}$ for event i∈{1,…,n}, and let $w_{i}$ represent the percentage of portfolio funds to be allocated on option *i*, with the total allocation summing to $\omega =w_{1}+\cdots +w_{n}$. Let $r_{j}=\left(r_{1j},\ldots ,r_{nj}\right)$ denote the cross-sectional *n*-dimensional vector of outcomes across one observational row of data. Over *T* data points this leads to *m* historical unique vectors $u_{k}=\left\{r_{j}\;|\;r_{j}\neq u_{l},\mathrm{for}\;\mathrm{some}\;j,\;\mathrm{and}\;\mathrm{any}\;l\neq k\right\}$ for k=1,…,m such that each $u_{k}$ is unique, with *m* bounded by either *T* or the maximum number of possible combinations $\lambda^{n}$, so $m=\min(T,\lambda^{n})$. Basically, the collection of $u_{k}$’s is a unique representation of the $r_{j}$’s with no duplicates. Let us also denote $\eta =\sum_{i=1}^{n}I\left(w_{i}\right)\leq n$ as the number of chosen options in the portfolio, where $I(w_{i})$ is the indicator function for the event $\left\{w_{i}>0\right\}$. Then the GEPO problem is defined as the following optimization program, generalized for different kinds of option strategies,

(23) $$\begin{array}{c} \mathrm{minimize}\;\;D_{KL}\left(R_{Q}\;∥\;U_{m}\right)=\log_{\lambda }\left(m\right)+\sum_{k=1}^{m}\left(\frac{g^{\left(k\right)}\left(0\right)}{k!}\right)\log_{\lambda }\left(\frac{g^{\left(k\right)}\left(0\right)}{k!}\right)\\ \mathrm{maximize}\;\;G\left(\omega \right)=\frac{1}{\eta }\sum_{i=1}^{n}I\left(w_{i}\right)p_{i}\log_{\lambda }\left(1+\omega \right)+\frac{1}{\eta }\sum_{i=1}^{n}I\left(w_{i}\right)q_{i}\log_{\lambda }\left(1-\omega \right)\;+\\ \;\frac{1}{\eta }\sum_{i=1}^{n}I\left(w_{i}\right)\varrho_{i}\log_{\lambda }\left(1+\alpha \omega \right)+\frac{1}{\eta }\sum_{i=1}^{n}I\left(w_{i}\right)\varsigma_{i}\log_{\lambda }\left(1-\beta \omega \right),\\ \mathrm{subject}\;\mathrm{to}\;\;\omega =w_{1}+\cdots +w_{n}\leq 1,\\ \;w_{i}\geq 0\;\;\forall \;i,\\ \;w_{i}=w_{j}=\eta^{-1}\omega \;\;\forall \;\left\{\left(i,j\right):I\left(w_{i}\right)=I\left(w_{j}\right)=1\right\}, \end{array}$$

for the *m*-state uniform distribution $U_{m}$ and *k*th-derivative at x=0 of probability generating function

(24) $$g\left(x;w_{1},\ldots ,w_{n}\right)=\frac{1}{T}\sum_{j=1}^{T}x^{\left\{k\;:\;\left(I\left(w_{1}\right)r_{1j},\ldots ,I\left(w_{n}\right)r_{nj}\right)=u_{k}\right\}}.$$

The last constraint in the optimization problem stems from the fact that joint entropy measures randomness strictly based on the inclusion or exclusion of a random variable. The joint entropy value does not change upon changes to non-zero percentages of asset allocation, so any non-zero weight $w_{i}$ contributes the corresponding marginal entropy from asset *i*, regardless of the magnitude of $w_{i}$. For this reason every asset included in the portfolio is assigned an equal weighting of $\eta^{-1}\omega$.

### 4.2. Risk-Adjusted Performance

Here we will use the risk-adjusted ratio for comparing growth rates of gambling portfolios introduced in our previous paper (Mercurio et al., 2020) [2], called the *Growth Rate Over UNiform Divergence (GROUND) ratio*. This ratio measures the expected growth rate of a portfolio, adjusted by its risk level—relative entropy with respect to the uniform distribution. Let $U_{m}$ be the *m*-state discrete uniform distribution. Then for chosen portfolio $R_{a}$ and minimum risk portfolio $R_{b}$ existing in the *m*-state event space, the GROUND ratio $\Gamma_{m}$ is defined as

(25) $$\begin{array}{cc} \Gamma_{m} & =\frac{E(G_{a}\left(\omega_{a}\right)-G_{b}\left(\omega_{b}\right))}{D_{KL}\left(R_{a}\;∥\;U_{m}\right)-D_{KL}\left(R_{b}\;∥\;U_{m}\right)}\\ & =\frac{E(G_{a}\left(\omega_{a}\right)-G_{b}\left(\omega_{b}\right))}{H\left(R_{b}\right)-H\left(R_{a}\right)}, \end{array}$$

where $G_{a}\left(\omega_{a}\right)$ is the growth rate of the chosen portfolio with weighting $\omega_{a}$, $G_{b}\left(\omega_{b}\right)$ is the growth rate of the minimum risk portfolio with weighting $\omega_{b}$, $D_{KL}\left(R_{a}\;∥\;U_{m}\right)$ is the relative entropy of the chosen portfolio with respect to $U_{m}$, $D_{KL}\left(R_{b}\;∥\;U_{m}\right)$ is the relative entropy of the minimum risk portfolio with respect to $U_{m}$, and H(·) is the Shannon entropy.

## 5. An Option Portfolio Selection Example with GEPO

### 5.1. Data

In this example, actual put and call option data is presented for 20 randomly selected equities from the S&P100 composite index using the Wharton Research Data Services (WRDS) from the Wharton School of the University of Pennsylvania, found at wrds-www.wharton.upenn.edu. Prices, volumes, expiration dates, and other essential option data is compiled from June 2012 to January 2018 (June 2012 is the earliest month available that contains data for all listed securities). Included in this data are the Greek parameters for options, which are described in detail in the Equity Options Reference Manual from TMX Group Montreal Exchange guides and strategies documentation (2020) [22]. The delta of each option, from the Greek parameters for options, effectively represents an estimated probability of landing in-the-money at expiry, and this parameter serves as the estimated success rates for the portfolio optimization here. For the purposes of this paper, we are only concerned with deltas closest to, but not less than 50%, to ensure every possible option yields a slight edge. Using these data archives, we are able to build historical weekly bull put spread and bear call spread options by selecting the buy-sell pairs that most closely resemble a 1:1 odds wager for each expiration date, creating 287 unique data points for each equity. Weekly credit spread outcomes versus historical strike prices are recorded and computed as follows,

(26) $$\begin{array}{cc} I\left(E_{P}>S_{P}\right)-I\left(E_{P}\leq B_{P}\right)+\left(E_{P}-M_{P}\right)/\left(S_{P}-M_{P}\right), & \;\;\;\mathrm{for}\;\mathrm{put}\;\mathrm{spreads}\;,\;\mathrm{and}\\ I\left(E_{P}>S_{P}\right)-I\left(E_{P}\leq B_{P}\right)+\left(E_{P}-M_{P}\right)/\left(B_{P}-M_{P}\right), & \;\;\;\mathrm{for}\;\mathrm{call}\;\mathrm{spreads}\;, \end{array}$$

where $E_{P}$ is the expiration price for the underlying equity in question, $S_{P}$ is the strike price for the sold option, $B_{P}$ is the strike price for the bought option, and $M_{P}$ is the midpoint of the two strike prices. The result is a value between +1 and −1 inclusive, on the continuous line, where positive 1 is awarded for an expiration price above the upper strike, −1 for an expiration price below the lower strike, and partial continuous returns between −1 and +1 for spreads expiring in the middle interval in between strike prices from the credit spread payoff function diagrams in Figure 6 and Figure 7. Using this historical data, we are able to empirically calculate the estimated relative entropy of each option. The selected credit spread options and their respective outcomes against strikes over June 2012 to January 2018 are presented below in Table 1.

Over the following calendar year 2018, there are 52 weekly equity option expiration periods, and estimated success probabilities for each option are defined as follows. For put options, the negative delta represents the probability of landing in-the-money for bought puts, and (1+delta) represents landing out-the-money for sold puts. For call options, the delta represents landing in-the-money for bought calls, and (1−delta) represents landing out-the-money for sold calls. The historical results summarized in Table 1 are used to evaluate the estimated relative entropy risk of each option, as well as the combined estimated relative entropy of the composite portfolio. The emulation here shows how GEPO performs against leading Kelly criterion methods for picking a portfolio of options for each week throughout 2018.

For illustrative purposes, let us examine this method applied to the second week, with option expiration dates 12 January 2018. Table 2 lists the details of the selected group of equity credit spreads built of the appropriate option pairs.

GEPO determines which collection of credit spreads to select and what percentage of portfolio funds to allocate to each, in order to build the optimal risk–reward credit spread portfolio.

Each potential portfolio has an expected growth rate, given the delta projections, and an estimated relative entropy with respect to the uniform distribution. The historical data contains T=287 data points for each option, so the maximum joint entropy that can possibly be exhibited is $\log_{3}\left(T\right)=5.1515$, i.e., uniform distribution with m=287 possible probability states. Therefore, a portfolio $R_{Q}$ has an estimated relative entropy of

(27) $$\begin{array}{cc} D_{KL}\left(R_{Q}\;∥\;U_{T}\right) & =\log_{3}\left(T\right)-H\left(R_{Q}\right)\\ \; & =5.1515-H(R_{Q}), \end{array}$$

for joint entropy $H(R_{Q})$.

### 5.2. Efficient Frontier and Portfolio Selection

In the portfolio selection problem, the efficient frontier refers to the set of the optimal portfolios that yield the greatest expected return for a defined level of risk, or equivalently the least risk of a defined level of expected return (the dual problem). The efficient frontier illustrates the risk-return trade-off for a given set of optimal portfolios. Here we show the analogous efficient frontier for portfolios with discrete returns, comparing the expected growth rates and risk levels (estimated relative entropy) of each efficient portfolio. For the same week of 12 January 2018, Figure 12 below plots the potential portfolios and their respective expected growth rates against their inherent risk profile, the estimated relative entropy with respect to the uniform distribution Uniform(*T*) to historical joint outcomes of the portfolio.

For the current season emulation, the Kelly criterion strategy chooses the portfolio $R_{K}$ that maximizes the expected growth rate. For week 2 this leads to the top right-most data point K = (4.5206, 0.0056), with estimated relative entropy of 4.5206 and expected growth rate of 0.0056. This portfolio consists of just one equity credit spread from Table 2: bull put spread on IBM, consisting of buying a put with strike 150 and selling a put with strike 152.5, betting on IBM to expire above 152.5 with a 50.5% probability of success, 31.2% probability of loss, and 18.3% of a partial return. According to our extended Kelly criterion conditions from Section 2.2, the optimal bet size here is 12%, and thus the chosen portfolio for week 2 is 12% of portfolio funds on [150, 152.5] < IBM, shown below in Table 3. This strategy disregards any concept of risk associated with the expected portfolio growth rate of G(ω)=0.0056.

Alternatively, GEPO chooses the optimal portfolio based on the risk–reward trade-off. For each of the weeks 1 to 52, portfolio selection is performed according to the following GEPO problem with n=20, α=−0.5 and T=287 (using logarithm base 3 here since we are dealing with three outcome states: 1, 0, and −1),

(28) $$\begin{array}{cc} \mathrm{maximize}\;\; & G\left(\omega \right)=\frac{1}{\eta }\sum_{i=1}^{n}I\left(w_{i}\right)p_{i}\log_{3}\left(1+\omega \right)+\frac{1}{\eta }\sum_{i=1}^{n}I\left(w_{i}\right)q_{i}\log_{3}\left(1-\omega \right)\;+\frac{1}{\eta }\sum_{i=1}^{n}I\left(w_{i}\right)\varrho_{i}\log_{3}\left(1+\alpha \omega \right),\\ \mathrm{subject}\;to\;\; & D_{KL}\left(R_{Q}\;∥\;U_{m}\right)=\log_{3}\left(m\right)+\sum_{k=1}^{m}\left(\frac{g^{\left(k\right)}\left(0\right)}{k!}\right)\log_{3}\left(\frac{g^{\left(k\right)}\left(0\right)}{k!}\right)\leq 2,\\ & w_{1}+\cdots +w_{n}\leq 1,\\ & w_{i}\geq 0\;\;\forall \;i,\\ & w_{i}=w_{j}=\eta^{-1}\omega \;\;\forall \;\left\{\left(i,j\right):I\left(w_{i}\right)=I\left(w_{j}\right)=1\right\}, \end{array}$$

for Uniform(*T*) distribution as the target distribution, and for the *k*th-derivative at x=0 of probability generating function with *T* data points,

(29) $$g\left(x;w_{1},\ldots ,w_{n}\right)=\frac{1}{T}\sum_{j=1}^{T}x^{\left\{k\;:\;\left(I\left(w_{1}\right)r_{1j},\ldots ,I\left(w_{n}\right)r_{nj}\right)=u_{k}\right\}}.$$

Although the Kelly criterion places the entire wager on the option (or options) with the greatest expected growth rate, GEPO diversifies the portfolio by distributing the percent allocation across multiple options according to the appropriate risk profile. For week 2, GEPO selects data point D = (1.5845, 0.0035), with estimated relative entropy of 1.5845 and expected growth rate of 0.0035. This corresponds to the optimal portfolio of the six credit spreads listed below in Table 4, with a total portfolio allocation of 9% (compared to the 12% allocated by the Kelly criterion).

Looking at the portfolio efficiency via the risk-adjusted GROUND ratio, the GEPO portfolio has a GROUND ratio of $\Gamma_{m}=\left(0.0035-0.0021\right)/\left(1.5845-0.05\right)=0.213\%$, more than twice as efficient as the Kelly criterion portfolio at $\Gamma_{m}=\left(0.0056-0.0021\right)/\left(4.5206-0.05\right)=0.078\%$.

The actual expiration prices that follow for week 2 are IBM at 163.14 (win), AIG at 60.97 (94% partial loss), MCD at 173.57 (win), ORCL at 49.51 (win), FB at 179.37 (win), and MRK at 58.66 (loss), for a total return of 3.2% in week 2. Therefore, the Kelly criterion strategy experiences a gain of 12% of portfolio balance in week 2, while the competing GEPO strategy gains 3.2%.

### 5.3. Comparison to the Kelly Criterion Over Time

**Figure 13:**
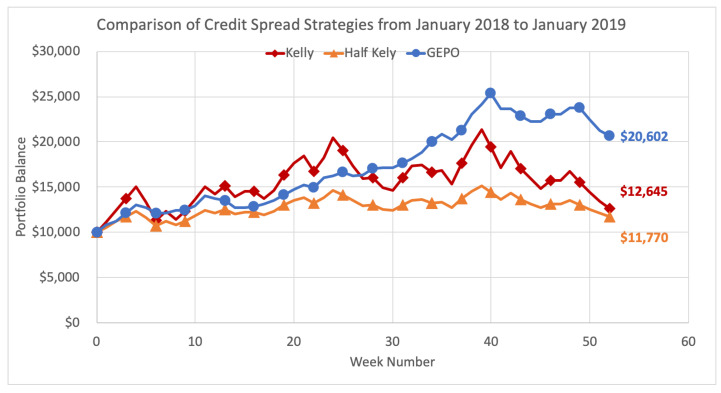
Comparison of Credit Spread Strategies from January 2018 to January 2020.

We demonstrate here the performance of GEPO versus the Kelly and Kelly variant strategies over the entire 2018 calendar year, executing option strategies at weekly intervals. Methods in the previous Section 5.2 are repeated week by week over the course of 52 weeks. The Kelly criterion strategy allocates the optimal investment size each week on the option (or options) that yield the greatest expected growth rate. Half Kelly is the same strategy but uses the fractional Kelly variant by allocating only half the Kelly criterion weighting on the same options. GEPO optimal risk strategy employs the GEPO algorithm each week to select the portfolio with the greatest growth rate subject to the main constraint that the portfolio has an estimated relative entropy of no greater than 2. Each strategy begins the year with $10,000 and the total results are shown below in Figure 13.

With consistent, sustainable returns, GEPO ultimately outperforms both the Kelly and half Kelly methods over the 52-week period, and more than doubles the initial investment by the end of the year. As the Kelly strategies experiences gross variability with the see-saw pattern returns, the diversification strategy of GEPO holds strong and consistently returns profits month after month. The main purpose of GEPO is to mitigate risk of inaccurate predictions, and goal is well accomplished. In the end, GEPO finishes the year at a profit of $10,062, more than 100% ROI, while the Kelly criterion gives up most gains and only retains $2645 (26.45%) profit, with half Kelly finishing up $1770 (17.7%).

## 6. Conclusions

Presented here is a new entropy-based combinatorial approach to option strategy portfolio selection called generalized entropic portfolio optimization (GEPO). GEPO is the most general method of the entropic portfolio optimizations introduced in our research series. We extend the notorious Kelly criterion to accommodate multiple assets and mixed returns, with direct application to option strategies. Using the convex risk measure relative entropy, GEPO presents a robust method for evaluating risk of option strategy portfolios and gives the mathematical tools to make data-driven portfolio selection decisions to mitigate risk. GEPO is robust, non-parametric, and indifferent to non-normality, asymmetry and small sample size data, making it an ideal approach to the option strategy portfolio selection problem. We show how GEPO comfortably outperforms leading Kelly criterion strategies in choosing optimal portfolios of equity credit spreads over 2018, both absolutely and in terms of risk-adjusted performance via the GROUND ratio. GEPO has a wide range of applications including option strategies such as covered calls, married puts, credit spreads, straddles, strangles, butterfly spreads, iron condors, and more.

## 7. Materials and Methods

Equity option data sourced from OptionsMetrics (www.optionmetrics.com), provided by Wharton Research Data Services (WRDS) from the Wharton School of the University of Pennsylvania, found at wrds-www.wharton.upenn.edu.

Data and R code (R version 3.5.1) used for the portfolio selection example demonstrated in this paper can be accessed from the following DropBox sharing links, Data: https://www.dropbox.com/s/nd6lowuz5ngpjuf/SP1002018.csv?dl=0, Code: https://www.dropbox.com/s/1v51xhako1jqkjh/SP1002018-GEPO.R?dl=0.

## Figures and Tables

**Figure 12:**
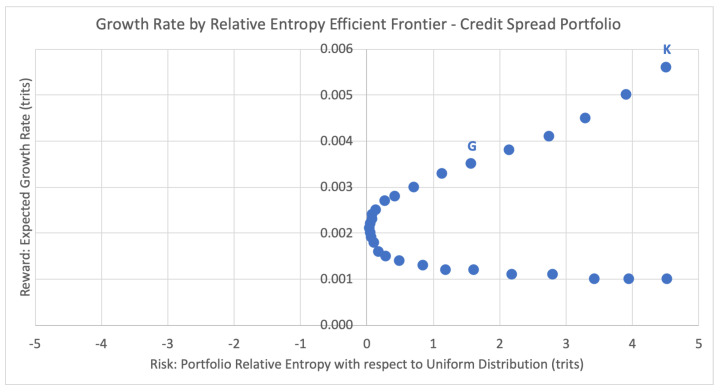
Growth rate by relative entropy efficient frontier.

**Table 1:** Mean outcome, average state probabilities and estimated relative entropy (in trits) of select equity credit spread options from July 2012 to January 2018.

Company Name	Symbol	Mean Outcome	*p*-prob	*q*-prob	ϱ-prob	$D_{\mathrm{KL}}$
Apple Inc.	AAPL	0.10124	52.3%	42.9%	4.9%	0.226675
Accenture	ACN	0.146462	54.3%	38.1%	7.7%	0.183754
American Intl. Group	AIG	0.121123	49.8%	38.2%	11.9%	0.118573
Bank of America Corp	BAC	0.139333	49.6%	32.6%	17.8%	0.071421
Biogen	BIIB	−0.048327	51.4%	46.1%	2.4%	0.281129
Caterpillar Inc.	CAT	0.151175	53.7%	38.5%	7.8%	0.180714
Capital One Financial Corp	COF	0.013458	46.6%	45.3%	8.1%	0.16507
Costco Wholesale Corp	COST	0.177373	51.8%	37.3%	10.8%	0.135782
Cisco Systems	CSCO	0.340769	59.6%	25%	15.4%	0.141729
Facebook Inc.	FB	0.128127	53.5%	42.3%	4.2%	0.242447
Intl. Business Machines	IBM	0.148669	53.2%	38.7%	8.2%	0.173439
Intel Corp	INTC	0.143774	51.7%	35.5%	12.8%	0.115093
Johnson & Johnson	JNJ	0.290017	61.9%	32.6%	5.5%	0.251597
JPMorgan Chase & Co.	JPM	0.223986	58%	36.4%	5.6%	0.230925
MasterCard Inc.	MA	0.125993	53.3%	40.8%	5.9%	0.209407
McDonald’s Corp	MCD	0.160243	53%	37.7%	9.3%	0.157863
3M Company	MMM	0.18277	55.7%	35.7%	8.6%	0.17661
Merck & Co.	MRK	0.177165	54%	37.9%	8%	0.17795
Microsoft	MSFT	0.170696	55.3%	38.5%	6.2%	0.209972
Oracle Corp	ORCL	0.286192	60.1%	30.6%	9.3%	0.191343

**Table 2:** Selected equity credit spreads on 12 January 2018, with their respective spread intervals, deltas and state projections.

Symbol	Spread Type	Spread Interval	Sell Delta	Buy Delta	*p*-proj	*q*-proj	ϱ-proj
AAPL	Put	[167.5, 170]	−0.496757	−0.405426	50.3%	40.5%	9.1%
ACN	Call	[149, 150]	0.492771	0.447763	50.7%	44.8%	4.5%
AIG	Call	[60, 61]	0.489573	0.345599	51%	34.6%	14.4%
BAC	Put	[28.5, 29]	−0.497381	−0.401975	50.3%	40.2%	9.5%
BIIB	Put	[317.5, 320]	−0.495617	−0.448496	50.4%	44.8%	4.7%
CAT	Put	[149, 150]	−0.497975	−0.43738	50.2%	43.7%	6.1%
COF	Call	[92.5, 93]	0.498336	0.465034	50.2%	46.5%	3.3%
COST	Put	[182.5, 185]	−0.497485	−0.417452	50.3%	41.7%	8%
CSCO	Put	[36.5, 37]	−0.496195	−0.374409	50.4%	37.4%	12.2%
FB	Put	[170, 172.5]	−0.487722	−0.392952	51.2%	39.3%	9.5%
IBM	Put	[150, 152.5]	−0.494561	−0.311505	50.5%	31.2%	18.3%
INTC	Put	[44, 44.5]	−0.496887	−0.403415	50.3%	40.3%	9.3%
JNJ	Call	[142, 143]	0.499681	0.408484	50%	40.8%	9.1%
JPM	Call	[105, 106]	0.499166	0.435065	50.1%	43.5%	6.4%
MA	Call	[146, 147]	0.494829	0.433672	50.5%	43.4%	6.1%
MCD	Put	[170, 172.5]	−0.499101	−0.335054	50.1%	33.5%	16.4%
MMM	Call	[242.5, 245]	0.495376	0.398127	50.5%	39.8%	9.7%
MRK	Call	[55, 55.5]	0.48691	0.405147	51.3%	40.5%	8.2%
MSFT	Call	[84.5, 85]	0.498834	0.451614	50.1%	45.2%	4.7%
ORCL	Call	[50, 51]	0.490397	0.374001	51%	37.4%	11.6%

**Table 3:** The Kelly criterion portfolio of options with percent allocation for expiration week 2, 12 January 2018.

Symbol	Spread Type	Spread Interval	*p*-proj	*q*-proj	ϱ-proj	Kelly Allocation %
IBM	Put	[150, 152.5]	50.5%	31.2%	18.3%	12%

**Table 4:** GEPO portfolio of options with percent allocation for expiration week 2, 12 January 2018.

Symbol	Spread Type	Spread Interval	*p*-proj	*q*-proj	ϱ-proj	GEPO Allocation %
IBM	Put	[150, 152.5]	50.5%	31.2%	18.3%	1.5%
AIG	Call	[60, 61]	51%	34.6%	14.1%	1.5%
MCD	Put	[170, 172.5]	50.1%	33.5%	16.4%	1.5%
ORCL	Call	[50, 51]	51%	37.4%	11.6%	1.5%
FB	Put	[170, 172.5]	51.2%	39.3%	9.5%	1.5%
MRK	Call	[55, 55.5]	51.3%	40.5%	8.2%	1.5%

## Abbreviations

The following abbreviations are used in this manuscript:

ADP	Approximate dynamic programming
AIG	American International Group
DEPO	Discrete entropic portfolio optimization
FB	Facebook Inc.
GEPO	Generalized entropic portfolio optimization
IBM	International Business Machines
KL	Kullback–Leibler
MCD	McDonald’s Corp
MRK	Merck & Co.
ORCL	Oracle Corp
REPO	Return-entropy portfolio optimization
WRDS	Wharton Research Data Services
